# Ammonia and beyond – biomarkers of hepatic encephalopathy

**DOI:** 10.1007/s11011-024-01512-7

**Published:** 2025-01-15

**Authors:** Juan-José Gallego, María-Pilar Ballester, Alessandra Fiorillo, Franc Casanova-Ferrer, Adrià López-Gramaje, Amparo Urios, Yaiza María Arenas, María-Pilar Ríos, Lucía Durbán, Javier Megías, Teresa San-Miguel, Salvador Benlloch, Paloma Lluch, Rajiv Jalan, Carmina Montoliu

**Affiliations:** 1 Fundación de Investigación Hospital Clínico Universitario de Valencia-INCLIVA, Valencia, 46010 Spain; 2 Departamento de Patología, Universidad de Valencia, Valencia, 46010 Spain; 3 Servicio de Medicina Digestiva, Hospital Clínico Universitario de Valencia, Valencia, 46010 Spain; 4 Servicio de Medicina Digestiva, Hospital Arnau de Vilanova, 46015 Valencia, Spain; 5 CIBERehd. Instituto de Salud Carlos III, Madrid, 28029 Spain; 6 Universidad Cardenal Herrera-CEU Universities, Valencia, 46115 Spain; 7 Liver Failure Group, Institute for Liver and Digestive Health, University College London, Royal Free Campus, London, UK; 8 European Foundation for the Study of Chronic Liver Failure (EF Clif), Barcelona, 08021 Spain

**Keywords:** Hepatic encephalopathy, Ammonia, Inflammation, Biomarker, Extracellular vesicles, Minimal hepatic encephalopathy, Microbiota, Neuroimaging, Brain injury markers

## Abstract

Ammonia is a product of amino acid metabolism that accumulates in the blood of patients with liver cirrhosis, leading to neurotoxic effects and hepatic encephalopathy (HE). HE manifestations can range from mild, subclinical disturbances in cognition, or minimal HE (mHE) to gross disorientation and coma, a condition referred to as overt HE. Many blood-based biomarkers reflecting these neurotoxic effects of ammonia and liver disease can be measured in the blood allowing the development of new biomarkers to diagnose cirrhosis patients at risk of developing HE. The effect of ammonia on the brain is modulated by severity of systemic inflammation, and both hyperammonemia and inflammation can induce oxidative stress, which may mediate the neurological alterations associated to HE. This review aims to provide the latest evidence on biomarkers of HE beyond ammonia. We present different approaches to predict overt HE based on the combination of blood ammonia with some analytical and clinical parameters. Magnetic resonance analysis of brain images could also provide sensitive diagnostic biomarkers based on neuroimaging parameters. Some reports suggest that markers of systemic inflammation, oxidative stress, and central nervous system-derived components, may serve as additional biomarkers of HE. The involvement of extracellular vesicles and microbiota in the pathophysiology of mHE and HE has recently acquired importance and it would be interesting to explore their usefulness as early biomarkers of the disease. It is important to have a biomarker or a combination of them for early diagnosis of mHE to improve its treatment and prevent progression to overt HE.

## Introduction

Brain dysfunction occurs in about 30–80% of patients with cirrhosis and is referred to as hepatic encephalopathy (HE), which is a complex behavioural, neuropsychiatric disorder. Clinically, HE manifestations can range from mild, subclinical disturbances in cognition, emotional regulation, or behaviour (minimal HE [mHE]) to gross disorientation and coma, a condition referred to as overt HE (OHE) (Montagnese et al. 2022). Some of the clinical features of HE overlaps with other brain conditions such a neurodegeneration in the mild forms and with delirium in those with more acute forms. Worldwide, there are about 10 M people with decompensated cirrhosis and about 30–40% of these patients will be hospitalised with OHE each year (Casadaban et al. 2015; Tapper et al. 2019). In Europe, about 200 K people suffer from decompensated cirrhosis and it is estimated that about 65 K patients per year are hospitalised with OHE, with the consequent economic burden on health systems, in addition to the attendant loss of work force (Moon et al. 2020). Both the burden of HE is increasing, and also charges per hospital stay (Elsaid et al. 2020; Louissaint et al. 2022). Importantly, when patients with cirrhosis develop an episode of OHE, they are prone to repeated episodes, the chance of complete reversibility drops, their health-related quality of life declines and the risk of death is abruptly increased up to 20–30% at 1-year (Arguedas et al. 2003; Tapper et al. 2016).

Currently, mHE is diagnosed using tests such as psychometric hepatic encephalopathy score (PHES) or critical flicker frequency, which are time-consuming to perform and interpret. Consequently, they cannot be routinely applied in clinical practice. This highlights the need to identify biomarkers for mHE and OHE that would enable early diagnosis and improved treatment.

Biomarkers are measurable and objectively evaluable characteristics that indicate a normal or pathological biological process or the response to a therapeutic intervention (Biomarkers Definitions Working Group 2001). Their study has significantly advanced in recent decades due to technological progress, molecular biology, and omics approaches, as well as the integration of these fields. Biomarkers are of particular interest in clinical practice for detecting and monitoring various diseases. They can serve as tools for early diagnosis, disease severity stratification, prognosis assessment, and predicting therapeutic response (Mayeux 2004; Jain 2017). Furthermore, studying disease-specific biomarkers is valuable for improving the development of novel therapies and evaluating their efficacy within clinical trials (Rolan 1997; Biomarkers Definitions Working Group 2001).

In the spectrum of HE, the research on specific biomarkers holds special relevance from multiple perspectives. On the one hand, it is crucial for understanding the pathophysiology of the condition from its early stages and its progression to more severe phases, facilitating the development of therapeutic targets and early-phase clinical trials. On the other hand, the need to identify a reliable biomarker is even more critical in mHE, as these biomarkers could provide a valuable tool for the early diagnosis in a simple and rapidly applicable manner. This would enable earlier treatment, significantly enhancing its effectiveness in preventing progression to OHE. Even when OHE is already present, biomarkers could prove useful in these stages to assess disease severity or identify patients who would benefit from prophylactic measures.

Ammonia is a product of amino acid metabolism that is known to accumulate in the blood of patients with cirrhosis due to the reduced function of the urea cycle, which is uniquely located in the liver. In health, its circulating levels are tightly controlled. In liver disease, hyperammonemia is commonly observed and recognized to play a pivotal role in the pathogenesis of HE (Felipo and Butterworth 2002; Bosoi and Rose 2009). Therefore, emerging evidence supports the utility of ammonia for risk stratification. However, ammonia measurement is complex and requires careful sample handling, rapid transport to the laboratory in refrigerated conditions and the values obtained are highly variable making it difficult to compare across laboratories. Therefore, the role of ammonia in guiding HE treatment is still unclear and there is equipoise in its use in clinical practice.

Many blood-based biomarkers reflecting these neurotoxic effects of ammonia and liver disease can be measured in the blood allowing the development of new biomarkers to diagnose cirrhosis patients at risk. It is also clear that the effect of ammonia on the brain is modulated by severity of systemic inflammation, which is commonly observed in patients with cirrhosis (Albillos et al. 2014, 2022). Therefore, markers of systemic inflammation and neuronal function may serve as additional biomarkers.

The aim of the present narrative review is to provide the latest evidence on biomarkers of HE beyond ammonia. Tables 1 and 2 present a summary of possible biomarkers for hepatic encephalopathy based on different kind of parameters measured, which will be discussed in the following sections.

**Table 1:** Summary of possible biomarkers for hepatic encephalopathy based on different kind of parameters measured

Biomarker	Prediction	Comments	References
*Ammonia and analitycal parameters*			
Albumin	mHE	Low sensitivity	Demirciler 2023
AMM-ULN	Risk of OHE	> 1.4 times over normal	Tranah et al. 2022; Balcar et al. 2023
CCHE score	Covert HE (mHE + HE grade I)	Variables and formula: 8*1 (history of OHE = yes) + 12*1 (clinically detectable ascites = yes) + 2*(38—S– ANT1 score) + 1*(21– points in the activity domain of CLDQ) + 0.5*(50– serum albumin level [g/dL]) If S-ANT1 > 38 or serum albumin > 50 g/dL, insert 38 or 50 as a maximum in the formula, respectively	Labenz et al. 2019
BABS score	Risk of OHE	Variables: bilirubin and albumin levels and non-selective beta-blockers and statins use. Score calculation in Table 3	Tapper et al 2018
AMMON-OHE model	Risk of OHE	Variables: AMM-ULN, sex, diabetes, albumin, creatinin C-index: 0.844	Ballester et al. 2023; link: https://ammon-ohe.shinyapps.io/ammon-ohe/
Glutaminase gene	Risk of OHE	Variations in a microsatellite region in the promoter of Glutaminase gene associated with OHE	Romero-Gómez et al. 2010; Mayer et al. 2015
*Metabolic profile*			
Serum metabolites signature	mHE	Increased levels of glucose, lactate, trimethylamine-N-oxide, glycerol and methionine distinguish mHE patiens from those without	Jiménez et al. 2010
CSF metabolites signature	Severity of OHE	72 metabolites altered in OHE. carnitine, 5-hydroxyindoleacetic acid and uracil related to OHE severity	Weiss et al. 2016
*Inflammatory parameters*			
IL-6, IL-18	mHE	Twice in mHE vs non-mHE. IL-6 levels in mHE patients > 11 pg/mL	Montoliu et al. 2009
IL-6, IL-17, STAT3	mHE	Elevated in mHE	Luo et al. 2012; Wu et al. 2016; Gairing et al. 2022
IL-17	mHE	IL-17 levels in mHE patients > 49 pg/mL	Li et al. 2015
Systemic inflammation plus ammonia	mHE, OHE	Sinergistic effect of ammonia and inflammation	Shawcross et al. 2004; Shawcross et al. 2011; Felipo et al. 2012a, b; Montoliu et al. 2015
Increased pro-inflammatory cytokines and chemokines	mHE	IL-6, IL-21, IL-17, IFN-γ, IL-12, IL-18, TNF-α, IL-1β, IL-22, and IL-15, and chemokines CCL20, CXCL13, and CX3CL1 are increased in mHE	Mangas-Losada et al. 2017
IgG, IL-15, CXCL13, IL-6, CX3CL1	mHE	AUROC: greater than 0.75	Mangas-Losada et al. 2017
IL-6, IL-18, TNF-α	OHE	Elevated in OHE and correlated with their severity	Luo et al. 2013; Onal et al. 2011; Komala et al. 2020; Odeh et al. 2004; Goral et al. 2011
*Immunological parameteres*			
Pro-inflammatory intermediate monocytes	mHE	Increased CD14^++^CD16^+^ monocytes in mHE	Mangas-Losada et al. 2017
Activation of B and T-lymphocytes	mHE	Increased activated B and CD4^+^ T lymphocytes, and autoreactive CD4^+^CD28^−^ T lymphocytes in mHE	Mangas-Losada et al. 2017
Th22 and follicular Th lymphocytes	mHE	Expansion of Th22 and Thf lymphocytes in mHE	Mangas-Losada et al. 2017
*Oxidative and nitrosative stress parameteres*			
GSSG/GSH ratio, malondialdehyde	mHE, OHE	Reduced in mHE and OHE. Correlate with mHE cognitive and motor impairment severity	Görg et al. 2010; Gimenez-Garzó et al. 2015, 2018
Serum 3-nitrotyrosine	mHE	AUROC: 0.85–0.96; cutoff 14 nM, (83–85% specificity; 82.5–94% sensitivity)	Montoliu et al. 2011; Felipo et al. 2013; Salman et al. 2021
Plasma cGMP	mHE	Increased plasma cGMP in mHE	Montoliu et al. 2007
*Neurotransmission and neuroimaging parameters*			
P300 event-related potential latency	mHE	Latency prolonged in mHE patients. AUROC: 0.725; cutoff 413 ms (61.1% specificity; 81.8% sensitivity)	Santana-Vargas et al. 2022
Mismatch negativity area	mHE	Correlate with attention deficits in cirrhosis patients. Distinguish mHE patients at cutoff 67 µV/ms (77% specificity; 83% sensitivity)	Felipo et al. 2012a, b
Mean kurtosis	mHE	Cuttoff 0.74 (89% specificity; 86% sensitivity)	Sato et al. 2019
Grey matter volume	mHE	Right cerebellum: AUROC of 0.986 at cutoff 0.698 mm^3^ (100% sensitivity; 91% specificity)	García-García et al. 2017
		Right insula/frontal inferior operculum: AUROC: 0.98; cutoff: 0.566 mm^3^ (92% sensitivity; 95% specificity)	
Increase thalamic volume	mHE	AUROC: 0.827; cutoff: 1.339% (87.5% sensitivity; 52.9% specificity)	Tao et al. 2013
	OHE	Increased with the severity of OHE	Tao et al. 2013
Connectivity strength	mHE	Right middle temporal gyrus: AUROC: 0.710; cutoff: 0.220 (79,3% sensitivity; 64.6% specificity)	Qi et al. 2014
		Left superior frontal gyrus: AUROC: 0.682 cutoff: 0.201 (64.5% sensitivity; 52,2% specificity)	
		Medial prefrontal cortex: AUROC: 0.884; cutoff: 0.520 (81,5% sensitivity; 70,4% specificity)	
Cerebral metabolites quantified by ^1^H MRS	mHE and OHE	mI/Cr depletion: 80%−85% sensitivity mI/Cr, Cho/Cr and Glx/Cr correlate with HE severity	Ross et al. 1994; Geissler et al. 1997
	mHE	Increased Glu in anterior cingulate cortex in mHE	Meng et al. 2015
	mHE and OHE	mI/Cr, Cho/Cr and Glx/Cr ratios: Normalization after liver transplantation, Improvement after 3 months of lactulose treatment	Naegele et al. 2000; Jain et al. 2013
*Neuroinflammation and CNS—derived components*			
PS100-β	mHE	Serum levels correlate with PHES score	Duarte-Rojo et al. 2016
	OHE	Increased in OHE patients and correlates with severity. AUROC: 0.8; cut-off 0.13 ng/mL (63.6% specificity; 83.3% sensitivity)	Saleh et al. 2007; Manzhalii et al. 2022; Duarte-Rojo et al. 2016
Model: PS100-β, ammonia, procalcitonin, MELD, TIPS, sodium	OHE	AUROC of 0.765	Weiss et al. 2024
Plasma NfL	mHE	Plasma NfL increased in mHE and OHE. AUROC between 0.66 and 0.87. Correlated with disease severity	Labenz et al. 2021; Fiorillo et al. 2023
	OHE	Plasma NfL increased in OHE	Labenz et al. 2021;de Wit et al. 2024
Serum GFAP	mHE	Correlated with disease severity	Gairing et al. 2023
*Biomarkers based on extracellular vesicles*			
TNF-α and MHCII in plasma EVs	mHE	Correlated with the degree of cognitive impairment	Gallego et al. 2022
Neuron-derived EVs	mHE	Proportion of neuron-derived Evs was higher in the blood of mHE patients	Gallego et al. 2022

*mHE* minimal hepatic encephalopathy, *AMM-ULN* ammonia level upper limit of normal, *OHE* overt hepatic encephalopathy, *CCHE* clinical covert hepatic encephalopathy, —S– ANT1 simplified animal naming test, *CLDQ* chronic liver disease questionnaire, *BABS* bilirubin–albumin–beta-blocker–statin, *CSF* cerebrospinal fluid, *AUROC* area under the receiver operating characteristic, *GSSG/GSH* ratio of GSSG/GSH in %, ^1^H MRS proton magnetic resonance spectroscopy, *mI* myo-inositol, *Cr* creatin, *Cho* choline, *GLx* Glutamine and Glutamate, PS100-β protein S100- β, *PHES* psychometric hepatic encephalopathy score, *MELD* model for end-stage liver disease, *TIPS* transjugular intrahepatic portosystemic shunts, *NfL* neurofilament light chain protein, *GFAP* glial fibrillary acidic protein, *MHCII* major histocompatibility complex class II, *Evs* extracellular vesicles

**Table 2:** Summary of biomarkers depending on their capacity for diagnosis and risk and severity prognosis of hepatic encephalopathy

Biomarker	Reference value
*Diagnosis of mHE*	
CCHE score*	> 57.5 score: high risk. Score formula in Table 1
Serum metabolites signature	Increased levels of glucose, lactate, trimethylamine-N-oxide, glycerol and methionine distinguish mHE patients from those without
Serum IL-6	AUROC of 0.75 at 2.7 pg/mL cutoff (ss: 70%; sp: 76%)
Serum IL-17	IL-17 levels in mHE patients > 49 pg/mL
Serum IL-15	AUROC of 0.77 at 5.68 pg/mL cutoff (ss: 73%; sp: 73%)
Serum CXCL13	AUROC of 0.76 at 159.6 pg/mL cutoff (ss: 68%; sp: 81%)
Serum CX3CL1	AUROC of 0.75 at 0.77 ng/mL cutoff (ss: 70%; sp: 69%)
Serum IL-18	AUROC of 0.68 at 199.8 pg/mL cutoff (ss: 71%; sp: 57%)
Serum 3-nitrotyrosine	AUROC of 0.85–0.96 at 14 nM cutoff (ss: 82.5–94%; sp: 83–85%
P300 event-related potential latency	AUROC of 0.725 at 413 ms cutoff (ss: 81.8%; sp 61.1%)
Mismatch negativity area	67 µV/ms cutoff (ss: 83%; sp: 77%)
Mean kurtosis	0.74 cutoff (ss: 86%; sp: 89%)
Right cerebellum GMV	AUROC of 0.986 at 0.698 mm^3^ cutoff (ss: 100%; sp: 91%)
Right insula/frontal inferior operculum GMV	AUROC of 0.98 at0.566 mm^3^ cutoff (ss: 92%; sp: 95%)
Increase thalamic volume	AUROC of 0.827 at 1.339% cutoff (ss: 87.5%; sp: 52.9%)
Right middle temporal gyrus CS	AUROC of 0.710 at 0.220 cutoff (ss: 79,3%; sp: 64.6%)
Left superior frontal gyrus CS	AUROC of 0.682 at 0.201 cutoff (ss: 64.5%; sp: 52,2%)
Medial prefrontal cortex CS	AUROC of 0.884 at 0.520 cutoff (ss: 81,5%; sp: 70,4%)
Plasma NfL	AUROC between 0.66 and 0.87 at 12.6 pg/mL cutoff (ss: 61%; sp: 68%)
*Severity of mHE*	
IL-6 and IL-18	Positive correlation with mHE severity
Oxidative stress	Oxidized/reduced glutathione ratio, reduced glutathione levels, malondialdehyde, and 3-nitrotyrosine correlate with cognitive and motor coordination impairment
Plasma NfL	Correlation with PHES
TNF-α and MHCII abundance in EVs	Correlation with PHES
*Treatment efficacy*	
Metabolic syndrome	Patients with clinical signs of metabolic syndrome tend to have a poorer response
Serum IL-21, IL-15, IL-18 and T cells CD69^+^	Patient who will not respond lacked an increase in this parameters
Plasma NfL	Patients who will respond lacked an increase in plasma NfL levels
Basal ganglia network connectivity	Patient who will not respond had connectivity impairment in this network
*Risk of OHE*	
AMM-ULN	> 1.4 times over normal
BABS score	≥ 21 score: 1 and 5-years high risk. Score formula in Table 3
AMMON-OHE model	link: https://ammon-ohe.shinyapps.io/ammon-ohe/
Glutaminase gene	Variations in a microsatellite region in the promoter of glutaminase gene predispose to OHE
*Diagnosis of OHE*	
Increase thalamic volume	Increased with the severity of OHE
PS100-β	AUROC of 0.8 at 0.13 ng/mL cutoff (ss: 83.3%; sp: 63.6%)
Model: PS100-β, ammonia, procalcitonin, MELD, TIPS, sodium	AUROC of 0.765
Valeric acid	AUROC of 0.830
*Severity of OHE*	
Systemic inflammatory response syndrome score	Correlate with grade 3 and 4 of OHE
IL-6, IL-18, TNF-α	Positive correlation with OHE severity
PS100-β	Positive correlation with OHE severity
CSF metabolites signature	CSF carnitine, 5-hydroxyindoleacetic acid and uracil positive correlated to OHE severity
Plasma NfL and GFAP	Positive correlation with OHE severity
Thalamic volume	Increased with the severity of OHE

*mHE* minimal hepatic encephalopathy, *CCHE* clinical covert hepatic encephalopathy, *AUROC* area under the receiver operating characteristic, *ss* sensitivity, *sp* specificity, *GMV* grey matter volume, *CS* Connectivity strength, *NfL* neurofilament, *PHES* psychometric hepatic encephalopathy score, *MHCII* major histocompatibility complex class II, *OHE* overt hepatic encephalopathy, *CCHE* clinical covert hepatic encephalopathy, *AMM-ULN* ammonia level upper limit of normal, *BABS* bilirubin–albumin–beta-blocker–statin, *CSF* cerebrospinal fluid, PS100-β protein S100- β, *MELD* model for end-stage liver disease, *TIPS* transjugular intrahepatic portosystemic shunts

^*^For diagnosis of covert HE (mHE plus HE grade I)

### Ammonia and its metabolism

Ammonia concentrations in blood and brain are regulated by its synthesis and degradation. In the blood of cirrhotic patients, the primary source of ammonia is the deamination of glutamine by intestinal glutaminase (Damink et al. 2002). In the brain, this deamination occurs in neurons (Márquez et al. 2013). Therefore, glutaminase activity has been studied as a factor related to the development of OHE. For the first time, Romero-Gómez et al. (2010) described variations in a microsatellite region in the promoter of the glutaminase gene associated with OHE in the Spanish population. Specifically, the presence of two long alleles of this microsatellite (≥ 14 repeats; 198 to 210 base pairs) was associated with higher glutaminase activity and with the development of OHE. Although the association between long alleles and OHE is corroborated by another independent study in the Caucasian population (Mayer et al. 2015), these results were not replicated in the East Asian population, indicating that the genetic predisposition given to these microsatellites is not universal and must be validated for each population (Ahn et al. 2017).

Ammonia can be detoxified in the brain and muscle by incorporating it into glutamine via glutamine synthetase. In the liver and intestinal mucosa, glutaminase degrades glutamine to glutamate and ammonia, incorporated into the urea cycle for degradation. The ability to metabolize glutamine to glutamate and ammonia by the intestinal mucosa can be assessed through oral glutamine challenge (OGC). In cirrhotic patients, intestinal glutaminase activity is increased and correlates with mHE (Romero-Gómez et al. 2004), and OGC results in a significant increase in blood ammonia levels, which does not occur in control subjects or those with liver transplants (Oppong et al. 1997). Similarly, an altered response to OGC in patients with mHE is associated with an increased risk of developing OHE (Romero-Gómez et al. 2002). These studies indicate that, beyond blood ammonia levels, it is crucial to investigate different aspects involved in ammonia metabolism as risk factors for the development of OHE and their utility as biomarkers.

### Prediction models based on clinical parameters

As previously mentioned, ammonia levels in cirrhotic patients are useful for stratifying the risk of developing OHE. In patients with stable cirrhosis, a 1.4 times ammonia level upper limit of normal (AMM-ULN) or more has been shown to define the risk of future hospitalization with OHE (Tranah et al. 2022; Balcar et al. 2023). Integrating other biochemical and clinical data can enhance its utility as a biomarker for diagnosis and determinate the risk of developing mHE and OHE. Regarding biochemical parameters, albumin levels have been identified as potentially helpful in diagnosing mHE, although their sensitivity is low when considered alone (Demirciler 2023). In this context, the AMMON-OHE model has been developed, which includes variables such as AMM-ULN, sex, diabetes, albumin, and creatinine to identify the risk of developing the first episode of OHE (Ballester et al. 2023). The model achieved a C-index of 0.844 was validated with an external cohort, and is currently available for use in clinical practice (link: https://ammon-ohe.shinyapps.io/ammon-ohe/).

Other scores based on clinical parameters have been developed, such as the CCHE (Clinical Covert Hepatic Encephalopathy) (Labenz et al. 2019), and BABS (Bilirubin–Albumin–Beta-Blocker–Statin) scores (Tapper et al. 2018) (Tables 2 and 3).

**Table 3:** Construction of a Risk Score for HE with the BABS (bilirubin–albumin–beta-blocker–statin) score

Variable	Category	Points in Baseline-Data Model	Points in Longitudinal-Data Model
Beta-blocker	No	0	0
	Yes	7	8
Statin	No	0	0
	Yes	−9	−4
Total bilirubin (mg/dL)	< 0.5	−2	−2
	0.6– 1	−1	−1
	1.1– 1.5	0	0
	1.6– 2	1	1
	2.1– 2.5	2	2
	2.6– 3	3	3
	3.1– 4	5	5
	> 4	18	18
Albumin (g/dL)	< 2	37	33
	2.1– 2.5	28	24
	2.6– 3	19	16
	3.1– 3.5	9	8
	3.6– 4	0	0
	> 4	−12	−11

Adapted from Tapper et al. 2018

The CCHE score was created to predict covert HE (which comprises mHE and HE grade 1) in cirrhotic patients using variables such as serum albumin levels, clinically detectable ascites, a history of OHE, and scores from the simplified animal naming test and the activity subdomain of the Chronic Liver Disease Questionnaire (Labenz et al. 2019). The CCHE score generates two cutoff points, stratifying patients into low-risk (< 53.5), intermediate-risk (53.5 ≤ CCHE score ≤ 57.5), and high-risk (> 57.5) categories for developing covert HE (see formula in Table 1). The scoring system demonstrates good sensitivity, specificity, and positive and negative predictive values (90%, 91%, 85%, and 94%, respectively).

The BABS score was developed to stratify the risk of developing OHE using biochemical variables (bilirubin and albumin levels) and the patient medication use (non-selective beta blockers and statins) (Tapper et al. 2018) (Tables 2 and 3). Two predictive models were developed for this score, using baseline or longitudinal data. The baseline-data model stratifies the 5-year risk of HE into low (< −10), medium (−9 to 20), and high (≥ 21). A score ≤ −10 is associated with a 27% risk, while a score > −10 corresponds to a risk > 49%. The longitudinal-data model stratifies the 1-year risk of HE into low (< 0), medium (1–20), and high (≥ 21). A score ≤ 0 is associated with a 6% risk, while scores ≥ 1 correspond to a 25% risk.

### Biomarkers based in metabolic profile

Beyond ammonia metabolism, other metabolic pathways are disrupted during cirrhosis, mHE, and OHE, leading to changes in metabolite levels in both serum and cerebrospinal fluid (CSF). For example, severe OHE is associated with increased levels of aromatic amino acids (AAA) and methionine in CSF (Cascino et al. 1982).

Studies conducted to distinguish cirrhotic patients with or without mHE based on their serum metabolic signature, showed that mHE patients exhibited increased levels of glucose, lactate, and trimethylamine-N-oxide, primarily, along with elevated levels of glycerol and methionine to a lesser extent. In contrast, patients without mHE were characterized by elevated levels of low-density lipoprotein, choline, alanine, α-acid glycoproteins, valine, acetoacetate, isoleucine, leucine, and glycine. Based on these changes, they developed a model with sensitivity and specificity of 87% and 95%, respectively (Jiménez et al. 2010).

Other studies revealed alterations in 72 metabolites in CSF from patients with OHE, most of which were associated with ammonia metabolism, energy pathways, methylation pathways, and aromatic amino acids, along with an increase in bile acids acids (Weiss et al. 2016), related with glymphatic system, which has been shown to be impaired in OHE animal models (Hadjihambi et al. 2019; Hsu et al. 2021). From this study, carnitine, 5-hydroxyindoleacetic acid and uracil were identified as being related to the severity of OHE, as they showed positive and negative correlations with the West Haven score scale and Glasgow coma scale, respectively.

### Biomarkers based on systemic inflammation

Systemic inflammation is another consequence of liver cirrhosis and a significant factor in the development of OHE. It has been demonstrated that systemic inflammation can modulate the toxic effects of ammonia on the brain (Shawcross et al. 2004) and that high systemic inflammatory response syndrome score, rather than ammonia in the blood, correlate with grades 3 and 4 of OHE (Shawcross et al. 2011). These studies support findings from other research groups that suggest a synergistic effect of systemic inflammation and ammonia levels in inducing neurological dysfunction in chronic liver disease (Felipo et al. 2012b; Montoliu et al. 2015).

In the early stages preceding OHE, there is already involvement of the immune system (Yadav et al. 2016). The levels of interleukin (IL) 6 and IL-18 are more than twice in patients with mHE compared to patients without mHE (Montoliu et al. 2009). These levels correlate with the severity of mHE, and it was observed that patients with mHE had IL-6 levels exceeding 11 pg/mL (Montoliu et al. 2009). Other studies have also demonstrated that the levels of cytokines IL-6 and IL-17, as well as the factor STAT3, are elevated and independently associated with mHE (Luo et al. 2012; Wu et al. 2016; Gairing et al. 2022). Moreover, in patients with mHE, IL-17 levels in plasma exceed 49 pg/mL (Li et al. 2015). Subsequently, more detailed characterization of immunological changes in patients with mHE was conducted (Mangas-Losada et al. 2017). Patients with mHE showed an increase in pro-inflammatory intermediate monocytes (CD14^++^CD16^+^), activated B and CD4^+^ T lymphocytes, and autoreactive CD4^+^CD28^−^ T lymphocytes. These immunological changes promote a pro-inflammatory environment characterized by elevated serum levels of pro-inflammatory cytokines such as IL-6, IL-21, IL-17, IFN-γ, IL-12, IL-18, TNF-α, IL-1β, IL-22, and IL-15, as well as chemokines CCL20, CXCL13, and CX3CL1. There is also an expansion of Th22 and follicular Th lymphocytes and increased activation of Th17 lymphocytes (see Figure 6 in Mangas-Losada et al. 2017). In this study, serum levels of IgG, IL-15, CXCL13, IL-6, and CX3CL1 achieved diagnostic values with an area under the receiver operating characteristic (AUROC) greater than 0.75 (Mangas-Losada et al. 2017).

In patients with OHE, elevated levels of some of these cytokines have been observed and correlated with the severity of the OHE, such as IL-18 (Onal et al. 2011; Komala et al. 2020), TNF-α (Odeh et al. 2004; Goral et al. 2011), and IL-6 (Luo et al. 2013). These findings support the hypothesis that immune system alterations in cirrhotic patients are potential biomarkers for the progression of OHE.

### Oxidative and nitrosative stress

Both hyperammonemia and inflammation can induce oxidative stress, which may mediate the neurological alterations seen in mHE and OHE (Görg et al. 2013). The presence of oxidative stress has been demonstrated in the blood and brain of patients with mHE and OHE (Görg et al. 2010; Giménez-Garzó et al. 2015, 2018). Specific markers of oxidative stress in the blood of patients with mHE, such as the oxidized/reduced glutathione ratio, reduced glutathione levels, malondialdehyde, and 3-nitrotyrosine, correlate with the severity of mHE and with attention and motor coordination impairments in these patients (Montoliu et al. 2011; Gimenez-Garzó et al. 2015). Serum levels of 3-nitrotyrosine are a marker of oxidative stress. Under oxidative stress conditions, nitric oxide reacts with superoxide to produce peroxynitrite, which in turn reacts with tyrosine to form 3-nitrotyrosine (Reiter et al. 2000; Pietraforte et al. 2003; Pacher et al. 2007). Levels of 3-nitrotyrosine are independently associated with mHE (Felipo et al. 2013) and have shown good diagnostic value for mHE, with an AUROC of 0.96, establishing a cutoff point of 14 nM, achieving 83% specificity and 94% sensitivity (Montoliu et al. 2011). Subsequent studies confirmed the diagnostic value of 3-nitrotyrosine in mHE, with AUROC values of 0.85, and sensitivity and specificity percentages of 85% and 82.5%, respectively, at a cutoff point of 14.15 nM (Salman et al. 2021).

Nitrosative stress is also implicated in neuronal alterations in patients with OHE (Genesca et al. 1999). The activation of guanylate cyclase by nitric oxide is altered in the brains of subjects with OHE, leading to impaired cyclic guanosine monophosphate (cGMP) formation, which contributes to the deterioration of cognitive functions during liver failure and hyperammonemia (Corbalán et al. 2002; Erceg et al. 2005a, b). cGMP homeostasis is disrupted in patients with liver cirrhosis, evidenced by increased blood levels but reduced lymphocyte levels of cGMP (Rodrigo et al. 2004; Montoliu et al. 2005). The disturbance in cGMP homeostasis in both the brain and blood of cirrhotic patients suggests that these blood alterations may reflect brain changes and could be associated with mHE. Studies in this area demonstrated that both cGMP levels and nitric oxide-induced guanylate cyclase activation in lymphocytes are elevated in patients with mHE, correlating with the severity of the condition (Montoliu et al. 2007).

### Neuroinflammation and central nervous system-derived components

The central nervous system (CNS) is a privileged tissue, isolated from the rest of the body by BBB, making it difficult to access. Postmortem analysis of this tissue aids in understanding the neuropathology associated with OHE, but obtaining biopsy samples from patients is not workable. Due to this difficulty, the analysis of CNS-derived components has been proposed to study its pathological and physiological state, primarily in CSF and blood. Several studies demonstrate the presence of neuroinflammation in patients with OHE (Cagnin et al. 2006; Dennis et al. 2014). Ammonia levels and systemic inflammation mediate this neuroinflammation. Ammonia directly affects microglia (Zemtsova et al. 2011) and systemic inflammation increases the permeability of the BBB. This increased permeability allows the infiltration of immune cells and inflammatory factors into the central nervous system (Kebir et al. 2007; Reboldi et al. 2009; Huppert et al. 2010; Rochfort et al. 2014).

#### S-100-β

The protein-S-100-β (PS100-β) is synthesized by astrocytes and Schwann cells and has been proposed as a peripheral biomarker for BBB permeability (Kanner et al. 2003; Marchi et al. 2004). It has been shown that OHE patients had higher levels of PS100-β than those without OHE and correlate with severity (Saleh et al. 2007; Manzhalii et al. 2022). This protein could have diagnostic value for OHE, as levels starting from 0.13 ng/mL could diagnose mHE and OHE with a sensitivity and specificity of 83.3% and 63.6%, respectively, and an AUROC value of 0.8 (Duarte-Rojo et al. 2016). In other studies, a model was developed that included PS100-β, ammonia, procalcitonin, MELD score, presence of transjugular intrahepatic portosystemic shunt and sodium, which achieved the best predictive value for OHE with an AUROC of 0.765 (Weiss et al. 2024).

#### Neurofilament light chain protein (NfL)

In recent years, NfL has been suggested as a marker for neurological diseases, which can be analyzed in blood and CSF. NfL is a structural protein of axonal cytoskeletons, and its levels correlate with neuronal damage in neurological diseases since axonal damage leads to NfL release, crossing the BBB and detectable in blood (Gaiottino et al. 2013; Osborn et al. 2019; Khalil et al. 2024). Recently, several studies have demonstrated the association between blood NfL and mHE, where NfL levels are elevated in mHE patients, correlate with the severity of the pathology, and provide diagnostic value with an AUROC between 0.66 and 0.87 (Labenz et al. 2021; Fiorillo et al. 2023). In patients with OHE, NfL levels were also elevated compared to cirrhotic patients without OHE (de Wit et al. 2024).

#### Glial Fibrillary Acidic Protein (GFAP)

The diagnostic value of serum GFAP has also been studied in mHE. GFAP is the major protein of the astrocyte cytoskeleton and is established as a biomarker of astrocyte damage and activation (Abdelhak et al. 2022). Similar to NfL, serum GFAP levels are also independently associated with mHE and its severity (Gairing et al. 2023).

#### Extracellular Vesicles (EVs)

EVs are structures formed by a lipid bilayer, ranging from 50 to 1,000 nm, generated by most cell types under both normal and pathological conditions, containing proteins such as cytokines, enzymes, surface proteins like receptors or ligands, lipids, and different types of nucleic acids (Jan et al. 2019). These EVs are present in all biological fluids, and their composition is conditioned by the cell type and physiological state that generates them. This characteristic makes them a potential tool for diagnosing and treating various diseases (Chen et al. 2008; Kalluri and LeBleu 2020). In neurological diseases, the composition of EVs from neurons has been established as a potential biomarker, as these EVs would reflect the pathological state of neurons and could be analyzed in blood samples (Bellingham et al. 2012; Winston et al. 2019). Studies conducted in animal models have shown the involvement of these EVs in hyperammonemia and OHE (Izquierdo-Altarejos et al. 2020, 2022, 2023). Recently, the composition and differences in the cellular origin of EVs in the blood of mHE patients have been characterized, as well as their role in modulating the immune system associated with mHE (Gallego et al. 2022). This study demonstrated that EVs isolated from the plasma of mHE patients were enriched in pro-inflammatory factors such as TNF-α and MHCII, that correlated with the degree of cognitive impairment in patients. Additionally, it was found that the proportion of neuron-derived EVs was higher in the blood of mHE patients. These studies suggest that analysis of EVs composition and source could serve as a biomarker for mHE and its progression to OHE, but more detailed studies are needed to improve its diagnostic value.

### Neurotransmission

Magnetoencephalography studies have revealed that patients with OHE exhibit alterations in neural synchronization and coupling within the basal ganglia-thalamus-cortex circuit, which modulates motor activity (Timmermann et al. 2003). Hyperammonemia and neuroinflammation also negatively affect neurotransmission, leading to alterations in neuronal networks and function (Sancho-Alonso et al. 2022a, b), which could be detected using non-invasive methods. A method to evaluate neurophysiological activity is through the recording of event-related potentials, where latency represents the time required to process the stimulus and is related to cognitive impairment (Polich 2012). One of the most studied event-related potentials in mHE is the auditory P300 potential, the latency of which is prolonged in these patients (Saxena et al. 2001, 2002). In this context, auditory P300 event-related potentials is capable of diagnosis of mHE in cirrhotic patients with a 61.1% of sensitivity and 81.8% of specificity (AUROC: 0.725; cutoff: 413 ms) (Santana-Vargas et al. 2022). Cirrhotic patients with mHE showed a reduction in the wave area of other event-related auditory potential, the mismatch negativity, and this reduction correlated with attention deficits in these patients. Reduced mismatch negativity wave area was able to differentiate patients with mHE at a cutoff point of 67 μV/ms with 83% sensitivity and 77% specificity (Felipo et al. 2012a).

### Biomarkers based on magneting resonance imaging and proton magneting resonance spectroscopy

Structural and functional magnetic resonance imaging (MRI) techniques could also provide useful parameters for the early diagnosis of mHE in a sensitive and widespread manner worldwide. In tractography studies, alterations in the levels of water diffusion in the brain tissue were observed in patients with HE and mHE, which indicates a microstructural alteration of the white matter directly related to the cognitive impairment of the patients (Miese et al. 2006; Montoliu et al. 2014). Hyperammonemia seems to play an important role in the severity of these microstructural alterations, as they are accentuated after inducing hyperammonemia in patients through the ingestion of amino acids (Mardini et al. 2011). On this topic, mean kurtosis, a parameter derived from the degree of water diffusion, in the putamen was able to differentiate cirrhotic patients with and without mHE using a cutoff point of 0.74, with a sensitivity and specificity of 89% and 86%, respectively (Sato et al. 2019). This parameter also showed potential diagnostic value in other brain regions, though with lower diagnostic potential.

On the other hand, a decrease in the density of both white and grey matter was observed in patients with cirrhosis and mHE. This alteration was also related to the psychometric results of the patients (García-García et al. 2017), but it does not seem to disappear after liver transplant (Guevara et al. 2011). Changes in grey matter volume were found to have diagnostic value for mHE, specifically in grey matter volume in right cerebellum (AUROC of 0.986; 100% sensitivity and 91% specificity; cutoff of 0.698 mm^3^) and in the right insula/frontal inferior operculum cluster (AUROC of 0.983; 92% sensitivity and 95% specificity; cutoff of 0.566 mm^3^) (García-García et al. 2017).

In a more localized way, a similar decrease in the thickness of the cortex was observed, both in the precuneus and in the temporal cortex, this alteration being much more marked in patients with mHE than in patients with cirrhosis, but without cognitive impairment, or in healthy subjects (Montoliu et al. 2012). A similar deterioration was also observed in the grey matter of the thalamus of patients with cirrhosis caused by the hepatitis B virus; reaching the point of suggesting the use of this deterioration as a biomarker of disease progress (Lin et al. 2022). Tao et al. (2013) found that thalamic volume was greater in patients with mHE and OHE, and it increased with the severity of OHE. Additionally, the increase in thalamic volume was able to distinguish between cirrhotic patients without mHE and those with mHE, with an increase of 1.339%, achieving AUROC values of 0.827, with 87.5% sensitivity and 52.9% specificity.

In early cognitive alterations such as mHE, structural damage, although slight, may have diagnostic utility. In these situations, the detection of alterations in brain activation and functioning patterns may be more relevant and useful.

Regarding functional MRI (fMRI) studies, resting state-fMRI (rs-fMRI) showed that in HE and mHE patients, decreased functional connectivity of the default neural network was associated with increased ammonia levels and poorer performance on psychometric tests such as the PHES battery (Zhang et al. 2012; Qi et al. 2014; García-García et al. 2017). This decreased connectivity was accentuated in patients with mHE compared to those with cirrhosis but without mHE. This decrease in connectivity was much more pronounced in patients who have suffered episodes of OHE and may be present even after patients had recovered their cognitive functions (Chen et al. 2012, 2013).

Reduced connectivity was also observed in neural networks associated with attention and executive functions, such as the attentional, fronto-parietal and salience networks (García-García et al. 2017). In the case of attentional networks, it has even been possible to classify patients as mHE or non-mHE solely on the basis of the state of connectivity in certain regions of interest in these networks (Chen et al. 2014).

Other studies have analysed the level of synchronisation at the local voxel level in certain brain regions. In these cases, in patients with mHE, a lower level of homogeneity was observed in the cuneus, precuneus and left inferior parietal lobe. On the other hand, homogeneity was increased in the cerebellum and left parahippocampal gyrus. Using these homogeneity alterations, and using artificial intelligence-based classification techniques, Chen et al. (2016) were able to classify patients with and without mHE with an accuracy of 82.9% sensitivity and 81.3% specificity.

Cerebral metabolites quantified by Proton magnetic resonance spectroscopy (^1^H MRS) could serve as biomarkers to stratify cirrhotic patients according to HE severity. Ross et al. (1994) indicated that myo-inositol (mI) depletion can detect HE and mHE with a sensitivity near to 90%. Changes in cerebral myoinositol and glutamine (Gln)/glutamate (Glu) levels correlated with the severity of HE, though these changes have also been observed in patients without HE (Geissler et al. 1997; Meng et al. 2015). Meng et al. (2015) found increased Glu levels in mHE patients compared to patients without mHE and controls. They suggested that Glu levels could be a sensitive indicator to evaluate the severity of mHE in patients with cirrhosis.

Decreased mI/creatine (Cr) and choline (Cho)/Cr ratios and an elevated Gln and Glu (Glx)/Cr ratio-were found to normalize after liver transplantation, in parallel with the dissapearance of the neuropsychologic signs of minimal or overt HE (Naegele et al. 2000). Treatment with lactulose also ameliorate metabolic parameters measured by MRS after 3 months of treatment in mHE patients (Jain et al. 2013).

It would be useful to identify MR parameters that distinguish cirrhotic patients with and without mHE with high sensitivity and diagnostic specificity. Moreover, MR studies are complicated to analyse, as well as expensive, so for daily clinical practice, it would be useful to have biomarkers measured in blood or other biological fluids that indicate the brain alterations of patients.

### Gut microbiota-based biomarkers

Alterations in the gut-liver-brain axis seem to play a relevant role in the induction of HE (Bajaj et al. 2012, 2014). In cirrhotic patients, bacterial translocation occurs in 25–30% of patients (Cirera et al. 2001), and several mechanisms promote it: bacterial overgrowth (potentiated by decreased intestine motility), immunological changes due to liver cirrhosis, and increased intestinal permeability (promoted by systemic inflammation) (Guarner et al. 1993; Francés et al. 2007; Bellot et al. 2013). This translocation and dysbiosis triggers several pathways that induce the release of pro-inflammatory cytokines and nitric oxide (Francés et al. 2007; Wiest and Garcia-Tsao 2005; Tse 2017). This systemic inflammation, nitric oxide and components derived from the intestinal microbiota can contribute to the permeability of the BBB and neuroinflammation characteristic of OHE (Shahbazi et al. 2023). The gut microbiome is altered in patients with liver cirrhosis which may contribute to alterations in the immune system and cognition.

In stool samples from patients with OHE, a decrease in autochthonous taxa such as Lachnospiraceae, Ruminococcaceae, and Clostridiales XIV, and an increase in pathogenic taxa such as Staphylococcaceae, Enterobacteriaceae, and Enterococcaceae have been found in both saliva and stool samples (Bajaj et al. 2014, 2015). Bacterial metabolites such as methanol and threonine and species as *Stenotrophomonas pavanii* and *Methylobacterium extorquens* were positive associated to OHE (Iebba et al. 2018). Certain taxa have been associated with better cognitive function, while others are associated with poor cognitive function in patients with OHE (Bajaj et al. 2012, 2014). These data confirm the pivotal role of the intestinal microbiota in the pathogenesis of OHE.

In patients with mHE, changes associated with the intestinal microbiota have also been found, such as the presence of gut ammonia-increasing bacteria *Streptococcus salivarius* and low diversity, along with a reduction in beneficial autochthonous bacteria and an increase in pathogenic gram-negative bacteria (Zhang et al. 2013; Wang et al. 2019; Bajaj et al. 2020; Luo et al. 2023). Studies in animal models reveal that mice receiving fecal transplants from patients with mHE developed neuroinflammation and microglial activation (Liu et al. 2020). These studies demonstrate that the intestinal microbiota is involved in the early stages of HE (Luo et al. 2023) Therefore, the intestinal microbiota and its components could be evaluated as an early biomarker.

Bajaj et al. (2019) found that the relative abundance of Lactobacillaceae in saliva and feces was increased in patients with mHE, because of that, saliva samples could also be a valuable resource for microbiota analysis related to this pathology. In the same study, genera bacteria from the Lachnospiraceae family, such as *Ruminococcus* and *Clostridium XIVb*, were found to be more abundant in cirrhotic patients without mHE and were associated with better cognitive function. This could help distinguish cirrhotic patients with and without mHE. Similarly, other studies have linked the presence of bacteria from the Prevotellaceae family with a reduced risk of developing mHE (Bajaj et al. 2020). On the other hand, the presence of small intestinal bacterial overgrowth has been established as an independent risk factor associated with mHE (Gupta et al. 2010).

Bacterial metabolism-derived compounds have also been studied as diagnostic parameters for HE. Specifically, short-chain fatty acids (SCFAs) and tryptophan metabolism compounds were analyzed for this aim in both serum and fecal samples (Wang et al. 2023). In this study, multiple compounds in serum or feces were capable of distinguishing between patients with OHE, cirrhotic patients, and controls. Notably, serum levels of valeric acid could distinguish between patients with and without OHE with an AUROC of 0.830 (Wang et al. 2023).

There are certain discrepancies in the abundance of microbial taxa and metabolites observed in cirrhotic patients, likely due to the diagnostic methods for mHE and OHE and the etiology of cirrhosis. These studies highlight the potential utility of microbiota-derived parameters as diagnostic tools for mHE and OHE. However, these parameters need to be studied in greater depth, considering multiple clinical variables.

### Biomarkers of treatment efficacy

OHE represents an advanced stage, where treatment aimed at reversing the condition becomes challenging. Comparative studies conducted before and after liver transplantation have shown persistent neurological alterations in patients who experienced episodes of HE prior to transplantation. This suggests that OHE may cause irreversible brain dysfunction; even after hepatic function is restored through transplantation (Garcia-Martinez et al. 2011). In contrast, an earlier stage in the HE spectrum, such as mHE, is potentially reversible if diagnosed early and treated appropriately. One of the most widely used treatments for mHE is rifaximin. Rifaximin has been shown to reverse cognitive and motor impairments, reduce peripheral inflammation, and lower NfL levels in mHE patients. However, several studies have identified preexisting differences between mHE patients who respond to treatment and those who do not, such as the fact that patients with clinical signs of metabolic syndrome tend to have a poorer response to rifaximin compared to those without (Ballester et al. 2022).

Mangas-Losada et al. (2019) demonstrated that mHE patients who did not respond to rifaximin lacked an increase in the early activation marker CD69 in T lymphocytes, as well as elevated levels of IL-21, IL-15, and IL-18 in plasma prior to treatment. Similarly, Fiorillo et al. (2023) demonstrated that before rifaximin treatment, patients with mHE who experienced a reversal of cognitive and motor impairment had significantly lower levels of NfL compared to those in whom the impairment did not reverse. MRI studies also found that patients who did not respond to treatment had intra-network connectivity in the basal ganglia network (Casanova-Ferrer et al. 2024)*.*

**Fig. 1:**
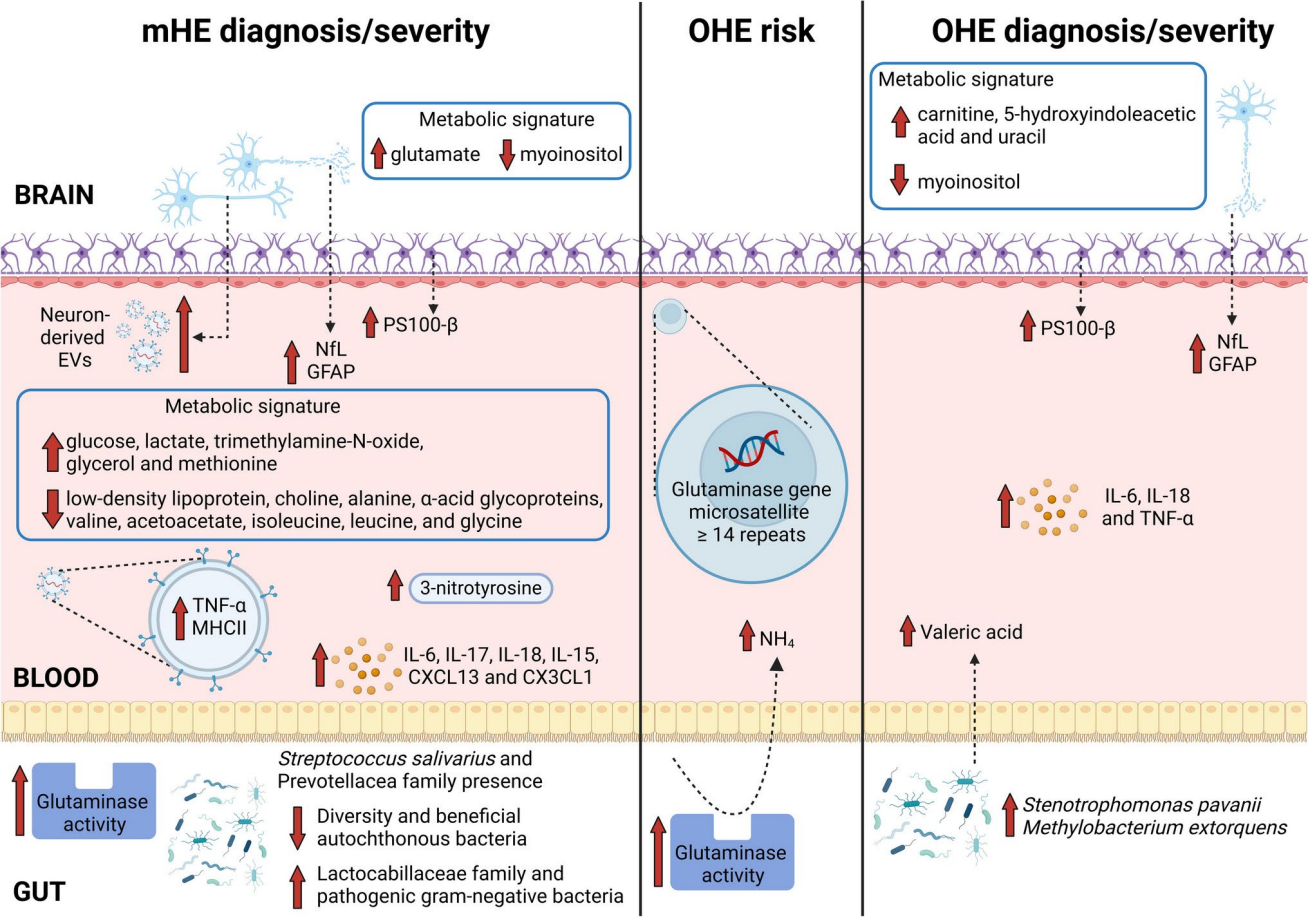
Summary of biomarkers from different origin as mHE and OHE diagnosis, risk stratification, and severity detection. See text for details explanation

Figure 1 shows a summary of biomarkers from different origin as mHE and OHE diagnosis, risk stratification, and severity detection.

## Conclusions

Biomarkers in the field of OHE have significant value as they could predict the onset of the disease or especially improve the early diagnosis of preclinical stages. This would allow for the anticipation of treatment and prevention of progression to more severe HE stages while also contributing to the development of novel therapeutic targets. Historically, ammonia levels have been considered a key factor in this pathology. However, when considered alone, ammonia has limited utility in diagnosing the various stages and progression of OHE. No studies currently compare the utility of ammonia against other biomarkers in both mHE and OHE, as well as their progression or severity. Such studies would be valuable for identifying the most effective biomarker. The development of predictive models that incorporate ammonia aims to enhance its predictive value by integrating additional variables involved in the pathophysiology disease. The most promising biomarkers in mHE and OHE would be these models, as they incorporate several variables relevant to the pathology. However, further research is still needed to develop and validate more specific models, tailored to the unique characteristics of individual patients and using accessible data in clinical practice. The benefit of using these models in the diagnosis of mHE lies in their ability to be applied quickly and objectively by medical professionals using biochemical and clinical history data. This would reduce the need for psychometric tests, which have disadvantages such as requiring active patient participation, being subject to evaluator bias, and demanding time for performance and interpretation, resources often unavailable in clinical settings. In addition to the study of blood molecules as biomarkers, another emerging field in recent years, particularly relevant for OHE biomarker research, is the evaluation of brain-derived molecules in peripheral blood. Although the initial data evaluating their role is interesting, further research is needed to better define their clinical usefulness. This approach offers a minimally invasive method to investigate biomarkers, providing valuable insights into neurological and systemic interactions without the need for invasive procedures. An easily measurable, rapid, and cost-effective biomarker for the diagnosis and prognosis of mHE and OHE, as well as for monitoring and predicting the respose to treatment, remains an unmet need. However, this work presents the most relevant information in this field, providing valuable insights for clinical professionals and researchers interested in utilizing, studying, or validating biomarkers for OHE in a clinical setting.

## Data Availability

Not applicable.

## Abbreviations

AAA: Aromatic amonio acids
AMM-ULN: Ammonia level upper limit of normal
AUROC: Area under the receiver operating characteristic
BABS: Bilirubin–albumin–beta-blocker–statin
BBB: Blood-brain barrier
CCHE: Clinical covert hepatic encephalopathy
cGMP: Cyclic guanosine monophosphate
CNS: Central nervous system
CSF: Cerebrospinal fluid
EVs: Extracellular vesicles
GFAP: Glial fibrillary acidic protein
HE: Hepatic encephalopathy
IL: Interleukin
MEG: Magnetoencephalography
mHE: Minimal hepatic encephalopathy
mI: Myo-inositol
MRI: Magnetic resonance imaging
^1^H MRS: Proton magnetic resonance spectroscopy
NfL: Neurofilament light chain protein
OGC: Oral glutamine challenge
OHE: Overt hepatic encephalopathy
PHES: Psychometric hepatic encephalopathy score
PS100-β: Protein S-100-β
